# S, N Co-Doped Graphene Quantum Dot/TiO_2_ Composites for Efficient Photocatalytic Hydrogen Generation

**DOI:** 10.1186/s11671-017-2101-1

**Published:** 2017-06-12

**Authors:** He Xie, Chengyi Hou, Hongzhi Wang, Qinghong Zhang, Yaogang Li

**Affiliations:** 1 State Key Laboratory of Modification of Chemical Fibers and Polymer Materials, College of Materials Science and Engineering, Donghua University, Shanghai, 201620 People’s Republic of China; 2 Engineering Research Center of Advanced Glasses Manufacturing Technology, College of Materials Science and Engineering, Donghua University, Shanghai, 201620 People’s Republic of China

**Keywords:** Graphene quantum dots, TiO_2_, Elemental doping, Photocatalysis, Hydrogen evolution

## Abstract

S, N co-doped graphene quantum dots (S,N-GQDs) coupled with P25 (TiO_2_) (S,N-GQD/P25) have been prepared via simply hydrothermal method. The as-prepared S,N-GQD/P25 composites exhibited excellent photocatalytic hydrogen generation activities, with a significantly extended light absorption range and superior durability without loading any noble metal cocatalyst. The photocatalytic activity of this composite under visible light (*λ* = 400–800 nm) was greatly improved compared with that of pure P25. This remarkable improvement in photocatalytic activity of the S,N-GQD/P25 composites can be attributed to that S,N-GQDs play a key role to enhance visible light absorption and facilitate the separation and transfer of photogenerated electrons and holes. Generally, this work could provide new insights into the facile fabrication of photocatalytic composites as high performance photocatalysts.

## Background

Hydrogen energy is a new green pollution-free energy with many advantages including high calorific value, easy storage and transportation, no pollution, etc. Given that water and sunlight are two of the most abundant and easily accessible sources in the real world, transferring the solar energy into H_2_ from aqueous solution has become a hot research topic in the field of photocatalysis and hydrogen energy. Compared with CdS, SiC and many other semiconductors those have been widely used for photocatalytic H_2_ evolution [1–6], TiO_2_ has several advantages, such as low cost, non-toxicity, good photochemical stability and long service life, which benefits its industrial applications [7]. However, the large bandgap (3.2 eV) of TiO_2_ and fast recombination of photogenerated electrons and holes restrict its solar energy conversion efficiency [8]. Massive strategies have been taken to solve this problem, such as doping with metal elements [9, 10], depositing with noble metal [11] sensitizing with organic dyes [12, 13] and so on. Recently, a great deal of interest has been attracted on TiO_2_-based composites with combining metal-free carbon materials, such as graphene and carbon nanotubes (CNTs), which could efficiently enhance photocatalytic activity due to the superior charge transport properties to reduce the recombination rate of photogenerated electron-holes. For example, Du et al. [14] has reported a photocatalysis based on graphene/TiO_2_ core–shell nanoparticles, and the enhanced photocatalytic activity was associated with the large extended photoresponsive range and high electron–hole separation efficiency due to the synergetic interactions among TiO_2_ and graphene material. However, graphene is intrinsically a semimetal with a zero bandgap, which considerably impedes its application in photocatalysis [15]. Besides, graphene as well as CNTs absorb a wide range of light, therefore may block other photocataysis from light irradiation [16]. Above drawbacks limit the photocatalytic performance of graphene- and CNTs-based composite photocataysis.

Graphene quantum dots (GQDs), as a new rising carbon nanomaterial, consist of few layers of graphene with a lateral dimension less than 10 nm and process unique properties derived from graphene [17]. Compared with traditional semiconductor quantum dots, such as ZnO [18], CdSe [19] and so on, GQDs exhibit higher water solubility, better chemical stability, low toxicity, excellent biocompatibility and photoelectrical properties. Therefore they have attracted a wide range of interests in sensing [20, 21], solar cells [22–24], bio-imaging [25, 26] and photocatalysis [27–30]. Recently, Qu et al. [31] has prepared GQD/TiO_2_ nanotube (GQD/TiO_2_ NT) composites by a simple hydrothermal method at low temperature. The photocatalytic activity of prepared GQD/TiO_2_ NT composites on the degradation of methyl orange (MO) was significantly enhanced compared with that of pure TiO_2_ nanotubes. Sudhagar et al. [32] has prepared GQDs/TiO_2_ hollow nanowires (HNW) architecture electrode for enhancing the light harvesting efficiency and the catalytic activity for water oxidation, without the need of sacrificial agents and demonstrated the underlying mechanism of photocarrier (e^-^/h^+^) transfer characteristics at GQDs/metal oxide interface during operation. Though there have been several reports suggesting the potential of GQDs as visible-light-driven photocatalysts, the lack of emission under long wavelength excitation and broad absorption in the visible region (*λ* > 400 nm) of GQDs still call for optimized methods [33]. Recently, nitrogen and sulfur co-doped graphene quantum dots (S,N-GQDs) are studied due to their broad photoabsorption in wide spectral range, high carrier transport mobility and excellent chemical stability. Qu et al [34] has demonstrated that S,N-GQDs processed much better absorption of visible light than pure GQDs and multicolor emission under visible light excitation. These results indicate that elemental doping of GQDs could produce promising catalysts for solar photocatalysis. Further researches should focus on the modification of GQDs to regulate the bandgap, broaden the photo absorption region, and improve photo-quantum efficiency. But major challenges remain in developing low-cost, stable, and highly active GQD-based photocatalysts.

In this paper, we reported a hydrothermal method for simultaneously synthesizing and doping GQDs with S and N. We further prepared the S,N-GQD/TiO_2_ (P25) composites by a facile hydrothermal route. This composite showed an excellent photocatalytic performance in H_2_ production from methanol aqueous solution under UV-vis irradiation without the assistance of any noble metal cocatalysts. The photocatalytic activities of S,N-GQD/TiO_2_ with different S,N-GQD loading amounts were also investigated. Finally, the mechanism for the improvement of photocatalytic performance was discussed based on experimental results.

## Methods

### Synthesis of the S,N-GQDs

The detailed synthesis process of S,N-GQDs has been reported elsewhere [35]. Typically, 1.26 g (6 mmol) citric acid and 1.38 g (18 mmol) thiourea were dissolved in 30 mL DMF and stirred for several minutes to obtain a clear solution. Then the solution was transferred in a 50 mL Teflon lined stainless steel autoclave. The sealed autoclave was heated up to constant 180 °C for 8 h and cooled down to room temperature. The final product was collected precipitate by adding ethanol into the solution and then centrifuged at 10,000 rpm for 15 min.

### Synthesis of the S,N-GQD/P25 Composites

The S,N-GQD/P25 composites were obtained by a hydrothermal method. Typically, 0.5 g P25 and 5 mL S,N-GQD (2 mg mL^−1^) were added into 20 mL distilled water. The mixture was kept stirring for 4 h at room temperature to obtain a homogeneous suspension. After that, the suspension was transferred into a 40 mL Teflon-sealed autoclave and maintained at 150 °C for 6 h. Then the S,N-GQD/P25 composites were collected precipitate by centrifugation at 4000 rpm for 5 min. And finally the solid was dried in vacuum oven at 50 °C overnight. To investigate the effect of the S,N-GQD content on the photocatalytic H_2_ evolution rate, the S,N-GQD/P25 composites with different contents of S,N-GQD (0, 1, 2, 3, 5, 8 and10 wt%) were prepared.

### Characterization

Transmission electron microscopic (TEM) and high resolution TEM (HRTEM) images were obtained by a JEOL JEM-2100 F microscope operating at 200 kV; X-ray diffraction (XRD) pattern were recorded on a Rigaku D/max-2500 diffractometer with a nickel filtrated Cu Kα radiation operated at 40 kV and 300 mA; Fourier transform infrared (FTIR) spectra were performed using Nicolet 6700 (Thermo Fisher); Raman spectra were carried out by NEXUS670 (Thermo Nicolet Corporation); UV–vis absorption spectra were measured using a UV-vis spectrophotometer Lambda 950 (Perkin Elmer, USA).

### Photocatalytic Hydrogen Generation

Fifty milligrams of photocatalyst powders were dispersed in a 100 mL aqueous solution which contains 10 mL methanol as the sacrificial agent. The UV-light and visible-light irradiations were generated from a 300 W Xe lamp without and with a 400 nm filter, respectively. The amount of generated H_2_ was determined with an online gas chromatograph.

### Photoelectrochemical Measurements

The transient photocurrent responses were measured in an electrochemical workstation with a conventional three-electrode system: a Pt plate as the counter electrode, a saturated calomel electrode as the reference electrode, and the as-prepared sample was coated on the ITO substrate as the working electrode. Specifically, the working electrode was prepared by coating the slurry made of 0.05 g photocatalyst, 0.2 g polyethylene glycol (PEG20000), and 1.0 mL water onto ITO glass electrodes by the doctor blade method, with subsequent calcining at 450 °C for 30 min. The active surface area of the working electrode that exposed to the electrolyte was about 2 cm^2^ and the thickness of the coated layer was about 8 mm. The electrolyte was 0.5 M Na_2_SO_4_ aqueous solution. The light source was a 300 W Xe lamp.

## Results and Discussion

**Fig. 1:**
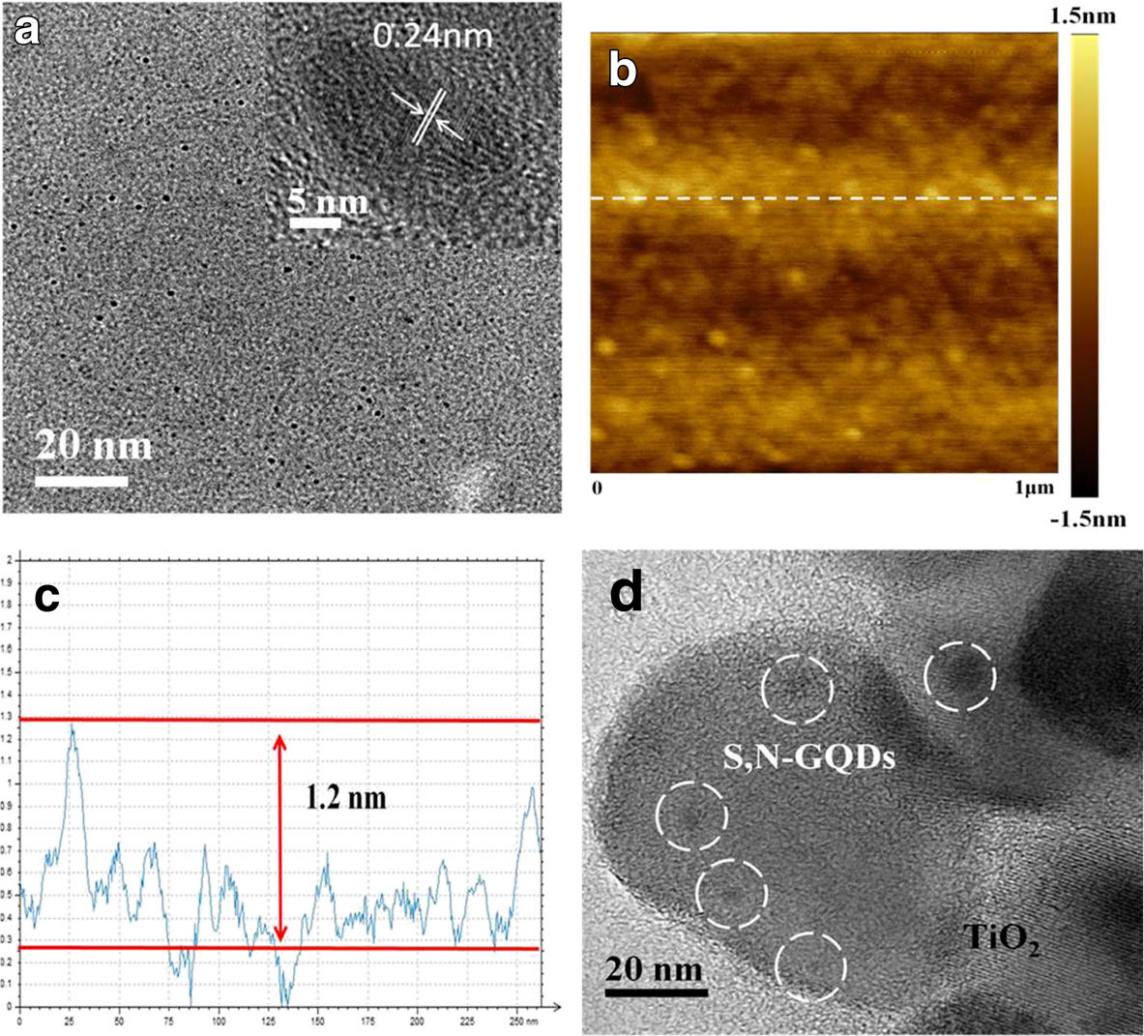
Morphology characterizations. **a** TEM and HRTEM images of the S,N-GQDs. **b**, **c** An AFM image and the height profile of the S,N-GQDs. **d** A TEM image of the S,N-GQD/P25 composites

Figure 1 shows the TEM images of the as-synthesized S,N-GQDs and S,N-GQD/P25 samples. The TEM images reveal that the S,N-GQDs have a uniform dispersion without apparent aggregation. In the HRTEM image in Fig. 1a, (0-110) lattice fringes with a spacing of around 0.24 nm for S,N-GQDs are visible [36], disclosing that the S,N-GQDs have a graphite nature. The atomic force microscopy (AFM) image and corresponding height-profile of S,N-GQDs are shown in Fig. 1b and c, respectively. The thickness of S,N-GQDs are mostly distributed in the range between 0.8–1.2 nm. After mixing with P25 nanoparticles, S,N-GQDs decorated on P25 and dispersed well, as revealed by the typical TEM image of the S,N-GQD/P25 composites (Fig. 1d).

**Fig. 2:**
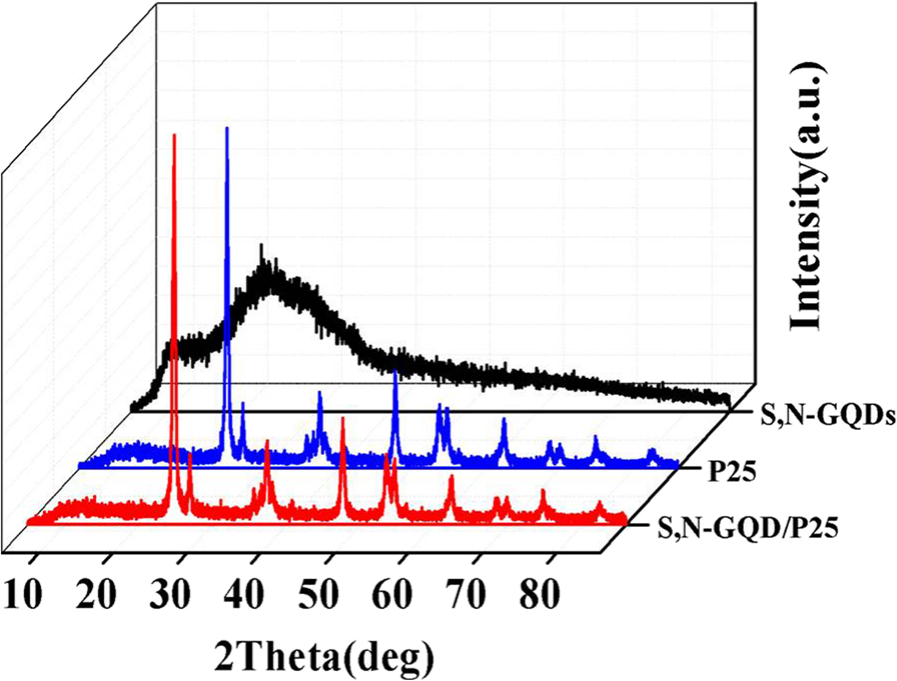
XRD patterns of the P25, S,N-GQDs, and S,N-GQD/P25 composites

The XRD patterns of pure P25, S,N-GQDs, and S,N-GQD/P25 composites are shown in Fig. 2. The P25 is a mixture of eighty percent anatase TiO_2_ and twenty percent rutile TiO_2_. The diffraction peaks at 25.28°, 36.96°, 37.8°, 48.05°, 53.89°, 55.02°, 62.69°, 70.26°, and 75.03° are attributed to (1 0 1), (1 0 3), (0 0 4), (2 0 0), (1 0 5), (2 1 1), (2 0 4), (2 2 0), and (2 1 5) plane of anatase TiO_2_; and the other peaks at 36.12°, 41.18°, and 56.72° are belonged to the (1 0 1), (1 1 1) and (2 2 0) plane of rutile TiO_2_ (JCPDS card No. 21–1272 and No. 21–1275). The spectrum of the S,N-GQDs shows one prominent peak at 25.6° that corresponds to the (0 0 2) planes of graphite structures (interlayer distance of ~0.34 nm) [37]. It is noteworthy that there are no typical peaks for S,N-GQDs can be found from the XRD spectrum of S,N-GQD/P25 and the location and intensity of the appeared peaks are barely changed compared to P25. This is due to the low content of S,N-GQDs in the composites, which clearly indicating the S,N-GQDs does not have an impact on TiO_2_ crystal structure and size.

**Fig. 3:**
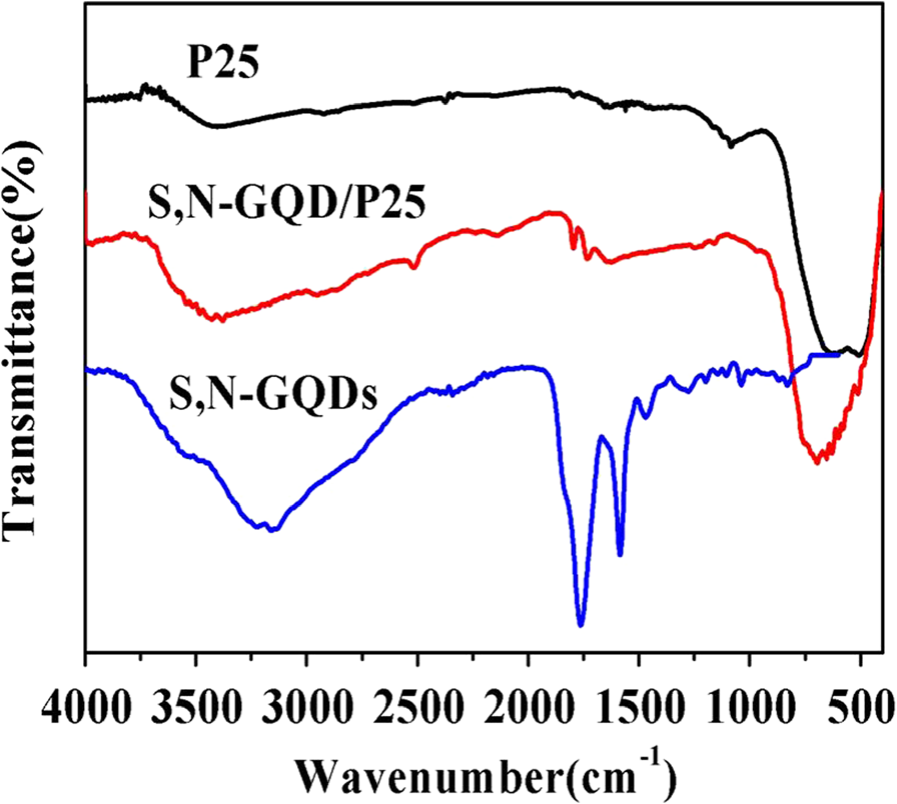
FTIR spectra of P25, S,N-GQDs, and S,N-GQD/P25

To demonstrate the successful loading of the S,N-GQDs on P25, we carried out FTIR and Raman spectrum measurements (Fig. 3). In the FTIR spectrum of S,N-GQDs, the O-H stretching vibration at 3232 cm^−1^; the vibrational peak of C = O at 1753 cm^−1^, asymmetric stretching vibrations of C = S and C–S at 1185 and 782 cm^−1^, respectively, and bending vibrations of N-H at 1558 cm^−1^ are visible. As for the pure P25, the abroad peak at 400–800 cm^-1^ corresponds to the bonds of Ti-O and Ti-O-Ti. Compared to the P25, this vibration band for S,N-GQD/P25 shows a slight red shift which is caused by the combination of S,N-GQDs and the stretching vibrations of Ti-O-C vibration. This confirms that the S,N-GQDs are coordinated with P25.

**Fig. 4:**
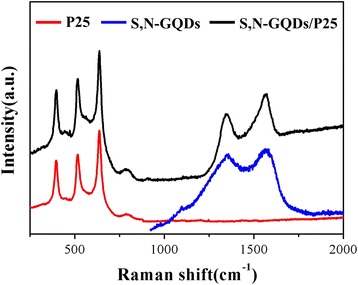
Raman spectra of P25, S,N-GQDs and S,N-GQD/P25

Figure 4 shows Raman spectra of P25, S,N-GQDs and S,N-GQD/P25. Three obvious characteristic peaks located at 396, 519 and 639 cm^−1^ can be ascribed to Raman active modes of P25 according to the symmetry group analysis. However, two additional D and G peaks located at 1357 and 1593 cm^−1^ can be seen in the S,N-GQD/P25 spectrum, which are the Raman-active modes of the S,N-GQDs. Based on all of the above results it can be concluded that the S,N-GQDs were successfully loaded onto the TiO_2_ nanoparticles.

**Fig. 5:**
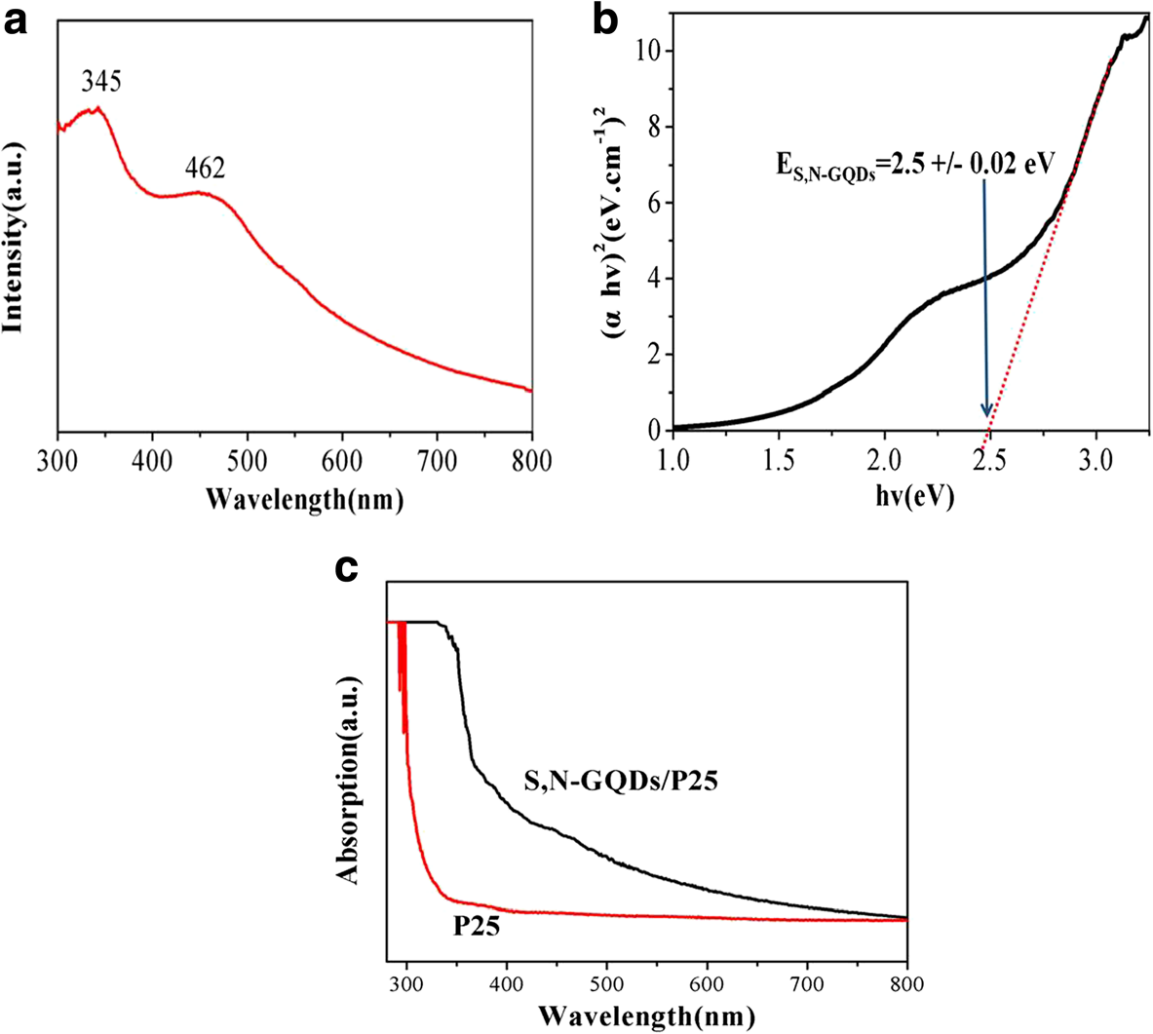
UV-vis measurements. **a** The UV-vis absorption spectrum of the S,N-GQDs. **b** The corresponding Tauc plot of the S,N-GQDs. **c** The UV-vis absorption spectra of the P25 and S,N-GQD/P25

Light absorption is a key factor that affects the photocatalytic performance of photocatalysts. The UV-vis absorption of S,N-GQDs (Fig. 5 a) displays two absorption bands centered at 345 and 462 nm, which is significantly different from the traditional GQDs with only one absorption band centered at around 340 nm [38–40]. It is evident that doping S and N into GQDs can change the band gap and result in this distinction. From the position of the absorption edge, the optical direct band gap values of the S,N-GQDs can be determined using the well-established Tauc’s relation (*αhυ)* ^*2*^ = *α*_0_(*hυ*-*E*_*g*_), where *hυ*, *α*_0_ and *E*_*g*_ are photon energy, a constant and optical band gap, respectively [41]. As shown in Fig. 5b, a gap energy of 2.5 eV for direct band gap for the S,N-GQDs can be easily obtained through the application of linear extrapolation. It is noted that the *E*_*g*_ of S,N-GQDs is lower than TiO_2_ (3.2 eV), giving this bandgap difference of 0.7 eV to make the S,N-GQDs be able to absorb and be excited by visible light [42]. The UV-vis absorption of the P25 and S,N-GQD/P25 composites measured in aqueous solution are shown in Fig. 5c. The pure P25 has almost no absorption in the visible light region of 400–800 nm, while the absorption of S,N-GQD/P25 composites extends to the visible range to 800 nm. Apparently, S,N-GQDs can efficiently broad the photo-response range of the S,N-GQD/P25 composites to visible light, which is expected to enhance its visible-light-driven photocatalytic activity.

**Fig. 6:**
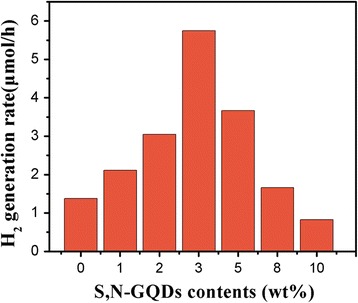
Photocatalytic H_2_ generation rates of pure P25 and S,N-GQD/P25 composites with different amounts of S,N-GQDs under UV–vis light

Figure 6 shows the photocatalytic performance of a variety of samples containing different amounts of S,N-GQDs (wt%) in S,N-GQD/P25 under UV-vis light irradiation in H_2_ production. It can be seen that pure P25 exhibits a relatively low photocatalytic H_2_ generation rate (1.7 μmol/h), probably due to that TiO_2_ can only absorb UV light and the rapid recombination of photogenerated electrons and holes. After the coupling with S,N-GQDs, the photocatalytic H_2_ generation rate of the composites increases gradually with the increase in amount of S,N-GQDs. The highest generation rate (5.7 μmol/h) is obtained in the 3 wt% S,N-GQD coupling sample, which is 3.6 times higher than that of pure P25. These results demonstrate that it is a feasible way to improve H_2_ generation activity of pure TiO_2_ by coupling it with the S,N-GQDs. This is mainly attributed to that there exists a good energy-band matching in the S,N-GQD-TiO_2_ heterojunction which facilitates highly efficient electron-hole separation at the interface [43]. In addition, The S,N-GQDs can efficiently transfer electrons and inhibit the recombination of photogenerated electrons and holes effectively. However, with further increasing the contents of the S,N-GQDs, the H_2_ generation rate gradually decreased, which is probably due to that the opacity and light scattering of the S,N-GQDs decreased the absorption of incident light and reduced catalytic active sites [44].

**Fig. 7:**
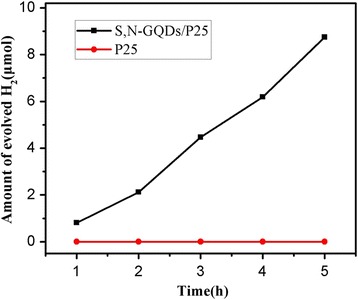
Photocatalytic H_2_ generation rates of pure P25 and S,N-GQD/P25 composites (3 wt.% S,N-GQD) under visible light

The photocatalytic H_2_ generation rate of the photocatalysts was also investigated under visible light (*λ* = 400–800 nm) irradiation. As shown in Fig. 7, pure P25 shows nearly no photocatalytic activity because it has almost no absorption within visible light (*λ* = 400–800 nm) due to its wide band gap (3.2 eV, it can only be excited by the light *λ* < 413 nm). On the contrary, with the loading of S,N-GQDs onto P25, the photocatalytic H_2_ evolution rate increases gradually under visible light irradiation, which reveals that the S,N-GQDs could be excited by visible light and possess photocatalytic activity.

**Fig. 8:**
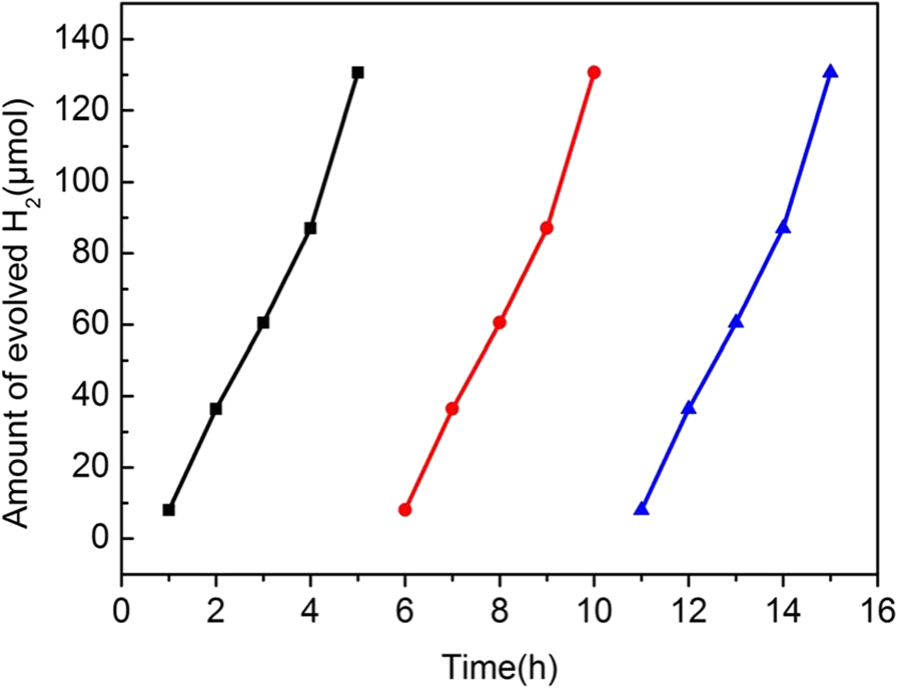
Three repeat cycle experiments of 3 wt% S,N-GQDs/TiO_2_

To further understand the practicality of S,N-GQD/P25 in the photocatalysis, we studied its cycle stability. Figure 8 reveals that the S,N-GQD/P25 composite photocatalyst has an excellent stability within three repeat cycles, indicating that the S,N-GQD/P25 could have a potential application in photocatalytic field.

**Fig. 9:**
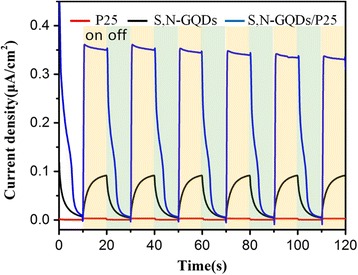
The transient photocurrent response of P25, S,N-GQDs, and S,N-GQD/P25 composites under UV–vis light irradiation

Furthermore, to get more information about the excitation and transfer of photogenerated charge carriers in photocatalysts, the transient photocurrent responses of P25 and S,N-GQD/P25 composite coated on ITO glass were investigated for several on-off cycles of UV-vis irradiation. As shown in Fig. 9, all of P25, S,N-GQDs, and S,N-GQD/P25 electrodes show sensitive photocurrent responses during repeated on/off cycles under the UV-vis irradiation. The changing trend of the photocurrent density is consistent with their photocatalytic H_2_ evolution activities. For the P25 electrode, there is a very weak photocurrent response to UV–vis light even at high applied potentials. For the S,N-GQDs electrode, the photocurrent response is stronger than that of the P25 alone, but becomes much slower. This photocurrent hysteresis behavior of the S,N-GQDs could result from high recombination rate of photogenerated electrons and holes and a high interfacial resistance between S,N-GQDs to charge transfer [45]. By contrast, after the combination of the S,N-GQDs, the photocurrent response of S,N-GQD/P25 has a notably improvement by nine times compared with P25 alone. The significantly enhanced photocurrent of S,N-GQD/P25 can be attributed to that S,N-GQDs is nanoscale fragment of graphene which can provide a larger active surface and greatly increase the contact area with the TiO_2_. Besides, S,N-GQDs can serve as the electron reservoir like frequently used co-catalyst Pt in photocatalytic H_2_, which is conducive to rapidly transfer photogenerated electrons. This result further proves that S,N-GQDs act as solid-state electron transfer reagent can accelerate the photogenerated electrons transfer, and indicates that S,N-GQD/P25 composite is a promising co-catalyst for photocatalytic H_2_ production.

**Fig. 10:**
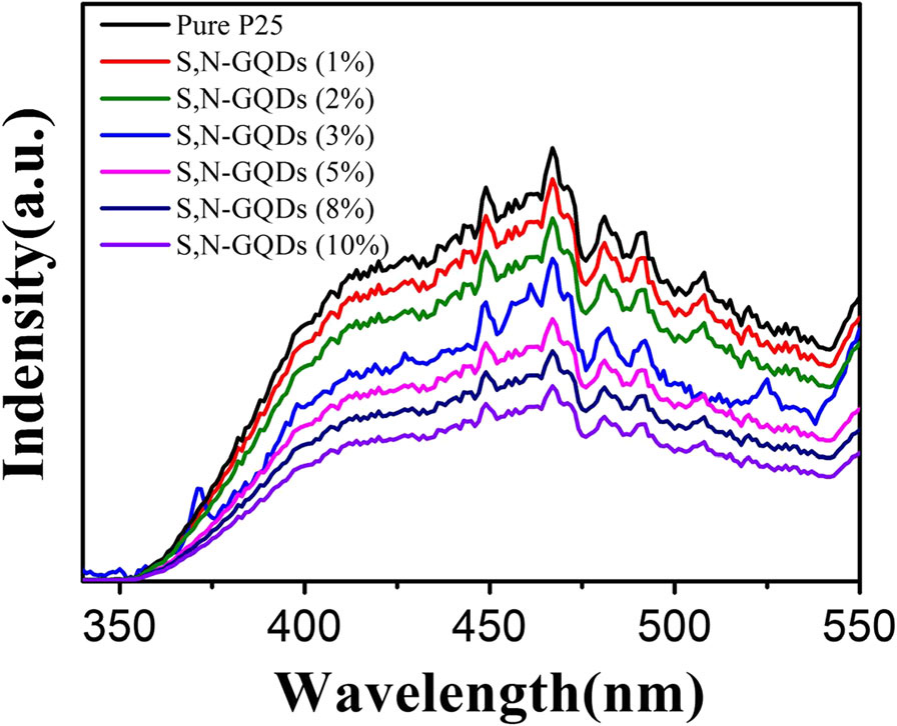
PL spectra of pure P25 and and S,N-GQD/P25 composites with different amounts of S,N-GQDs. Excitation wavelength: 280 nm

**Fig. 11:**
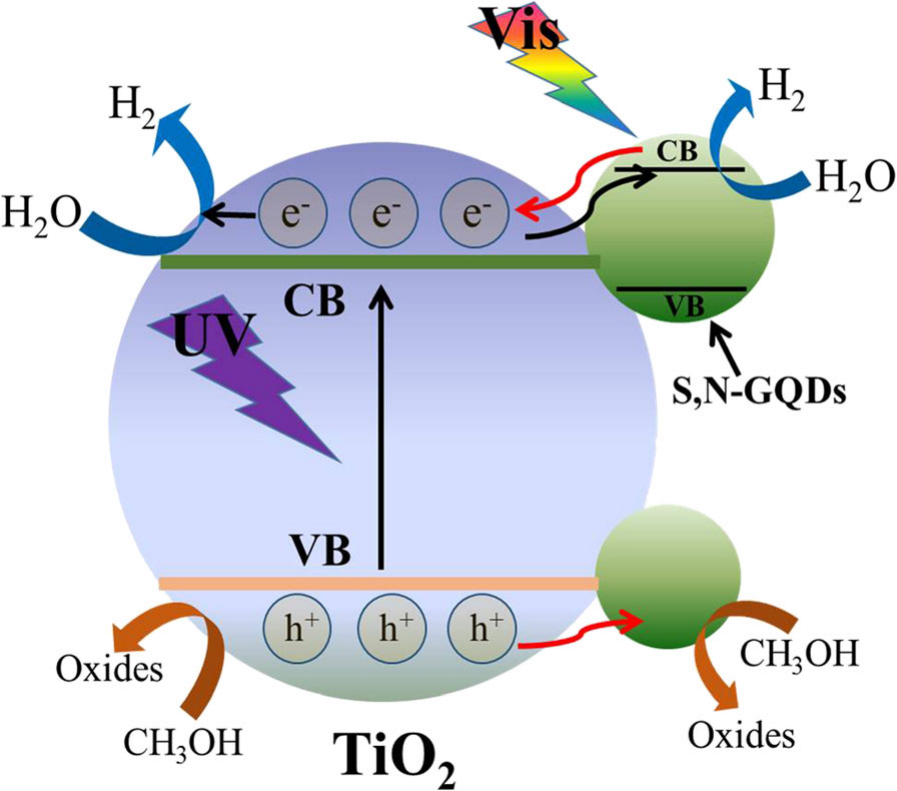
Proposed mechanism for the photocatalytic H_2_ evolution of S,N-GQD/P25 composites under UV-vis light irradiation

Furthermore, as shown in Fig. 10, the PL spectrum of pure P25 exhibits an emission band in the wavelength range of 350–550 nm, which was assigned to the excitonic band edge emission of TiO_2_. Compared with pure P25, all the S,N-GQD/P25 samples display a substantially decreased PL emission, and the quenching efficiency of PL emission increases with the increase of S,N-GQDs content. This observation reveals that charge recombination of TiO_2_ was greatly retarded by combination with S,N-GQDs. Based on above results, we proposed a possible mechanism for the enhanced photocatalytic H_2_ production activity of the S,N-GQD/P25 composites. As showed in Fig. 11, the mechanism can be described by the following three points: Firstly, under UV light irradiation, S,N-GQDs can serve as the electron reservoirs to trap photogenerated electrons from P25 and promote the separation of photogenerated electron-hole pairs efficiently, which is confirmed by PL measurement. Secondly, under visible light irradiation, the S,N-GQDs act as a photosensitizer to sensitize P25 and donate the electrons to the conduction band of P25, leading to the visible-light-driven photocatalytic H_2_ production activity. In addition, with a narrow bandgap of 2.5 eV, the S,N-GQDs can convert visible light and possess photocatalytic activity under visible light irradiation, which is confirmed by UV-vis absorption and photocatalytic H_2_ generation under visible light measurement. The whole photocatalytic reaction process can be described by the following equations [46]:

1 $$ \mathrm{photocatalyst} + h v\to\ {\mathrm{e}}^{\hbox{-} } + {\mathrm{h}}^{+} $$

2 $$ {\mathrm{h}}^{+} + {\mathrm{H}}_2\mathrm{O}\ \to \cdot p \mathrm{O}\mathrm{H} + {\mathrm{H}}^{+} $$

3 $$ {\mathrm{CH}}_3\mathrm{O}\mathrm{H} + \cdotp \mathrm{O}\mathrm{H}\ \to \cdot p {\mathrm{CH}}_2\mathrm{O}\mathrm{H} + {\mathrm{H}}_2\mathrm{O} $$

4 $$ \cdotp {\mathrm{CH}}_2\mathrm{O}\mathrm{H}\ \to\ \mathrm{H}\mathrm{CHO} + {\mathrm{H}}^{+} + {\mathrm{e}}^{\hbox{-} } $$

5 $$ 2{\mathrm{H}}_2\mathrm{O} + 2{\mathrm{e}}^{\hbox{-} }\ \to\ {\mathrm{H}}_2 + 2{\mathrm{OH}}^{\hbox{-} } $$

6 $$ \mathrm{Overall}\ \mathrm{reaction}:\ {\mathrm{CH}}_3\mathrm{O}\mathrm{H}\ \to\ \mathrm{H}\mathrm{CHO} + {\mathrm{H}}_2 $$

## Conclusions

In conclusion, we successfully prepared the S,N-GQD/P25 composites in aqueous solution. The composites were studied by TEM, HRTEM, FTIR, Raman and XRD analyses. Our results demonstrated that S,N-GQDs decorated on P25 can obvious broaden the visible light absorption of P25 and enhanced the activity on photocatalytic H_2_ production under UV–vis light irradiation. Especially, the 3 wt% S,N-GQD/P25 showed the best photocatalytic ability, which is about 3.6 times higher than that of the pure P25. Furthermore, the S,N-GQD/P25 composites also exhibited efficient photocatalytic H_2_ production activity under visible light, which won an advantage over P25. Overall, the S,N-GQD/P25 composites showed improved utilization of solar light for hydrogen production and energy conversion.
